# Non-surgical Endodontic Management of a Permanent Maxillary Canine With Vertucci Type 3 Canal Configuration: A Case Report

**DOI:** 10.7759/cureus.72565

**Published:** 2024-10-28

**Authors:** Paras M Gehlot, Brindha Murali, Susmita Ghosh, Shaima M Shafiq

**Affiliations:** 1 Conservative Dentistry and Endodontics, JSS Academy of Higher Education and Research, Mysuru, IND

**Keywords:** cone-beam computed tomography (cbct), maxillary canine, root canal anatomy, root canal therapy, vertucci type 3 canal configuration

## Abstract

To achieve success in endodontic treatment, it is essential to have a thorough understanding of the root and canal morphology. While maxillary canines typically have a single root and canal, studies have reported variations in different populations. This case report details the non-surgical endodontic management of a maxillary canine (tooth no. 13) with a Vertucci type 3 canal configuration in a 69-year-old female patient. The patient reported pain in the maxillary right canine (#tooth no. 13) and was diagnosed clinically with apical periodontitis. The patient's dental history revealed an ameloblastoma in the right mandibular region, successfully treated five years ago. Cone beam computed tomography aided the treatment. After completing the root canal treatment (#13) involving the two canals, an incidental loop-type accessory canal was noted. After three years of follow-up, the patient reported being asymptomatic. This paper reports the endodontic management of an uncommon type 3 canal configuration in the maxillary canine. Clinicians must remain aware of canal variations and utilize modern diagnostic tools like cone beam computed tomography to ensure all canals are located and adequately treated.

## Introduction

The maxillary and mandibular canines are essential from both an aesthetic and functional standpoint in the oral cavity. These teeth typically have single roots and single-rooted canals [1]. It is well established that numerous studies conducted worldwide have shown that different racial populations have distinct anatomical characteristics and related root canal morphology in their teeth [2]. Knowledge of root and root canal morphology is crucial for the success of both non-surgical and surgical endodontic treatments. Root morphology is complex and varies both internally and externally. To define the various canal configurations that are frequently encountered, several classifications have been proposed [3].

Although maxillary canines have fewer anatomical variations compared to other teeth, studies have reported different configurations in maxillary canines in different populations [1,4-6]. The root canal configurations of human permanent teeth, as classified by Vertucci, vary from a single canal to three separate and distinct canals [7].

Numerous studies agree that, in 75%-100% of cases, the type 1 Vertucci canal configuration is the most common in maxillary canines [1,6,8] The Vertucci Type 3 canal configuration starts as one from the pulp chamber, divides into two in the mid-root region, and then merges to exit as one apically [9]. This configuration is reported to vary from 0.7% to 11.6% [2,6,10].

Recently, cone beam computed tomography (CBCT) has been a successful tool in the field of endodontics to explore root canal anatomy. It has become popular because of its non-invasive nature and less radiation exposure compared to conventional CT [6,11]. This case demonstrates non-surgical endodontic management of a maxillary canine with Vertucci Type 3 canal configuration and an accessory canal using CBCT as a diagnostic tool.

## Case presentation

A 69-year-old female patient with no relevant medical history presented to the outpatient department with a chief complaint of pain concerning the right maxillary canine, which was aggravated while chewing food. Clinical examination revealed deep proximal caries involving the mesial surface of the maxillary right canine (#13). The pulp sensibility test (Cold and Electric pulp test) was negative, and the tooth was tender on vertical percussion. Past dental history revealed that the patient had undergone treatment for ameloblastoma in the right mandibular region five years ago.

**Figure 1:**
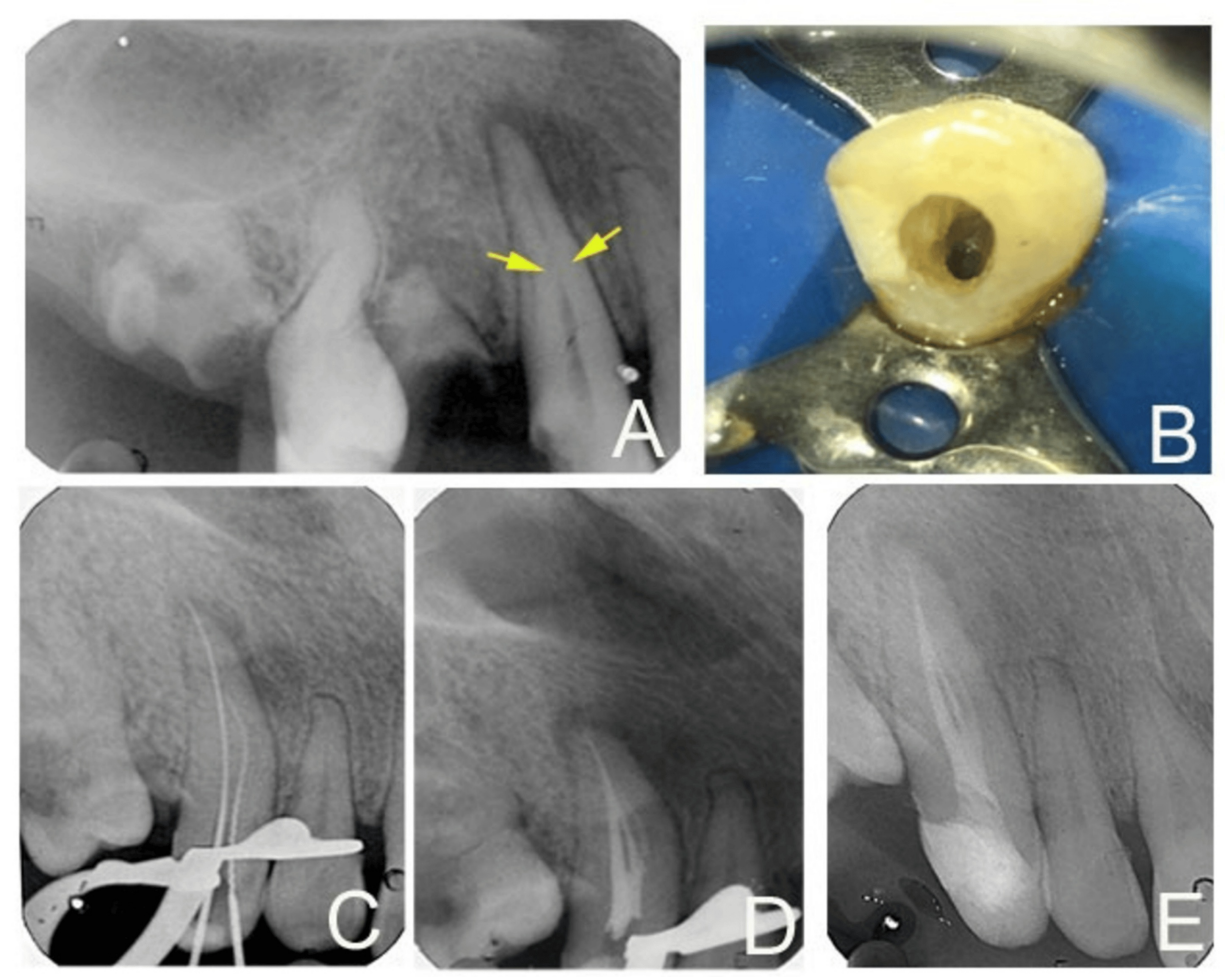
Radiograph and access cavity preparation (A) Pre-operative intra-oral periapical radiograph (IOPAR) (yellow arrow indicating “fast break”). (B) Access cavity under rubber dam. (C) Working length estimation IOPAR. (D) Immediate post-obturation IOPAR. (E) Post-operative radiograph after access restoration (with mesial shift).

The preoperative intraoral periapical radiograph (IOPAR) revealed mesial proximal caries in the crown, suggestive of close proximity to the pulp. A single canal root radiolucency originating from the pulp chamber disappears in the middle third and is further visible in the apical third (“fast break” appearance) (Figure 1A). An apical radiolucency (20 x 10 mm) was evident on the radiograph.

**Figure 2:**
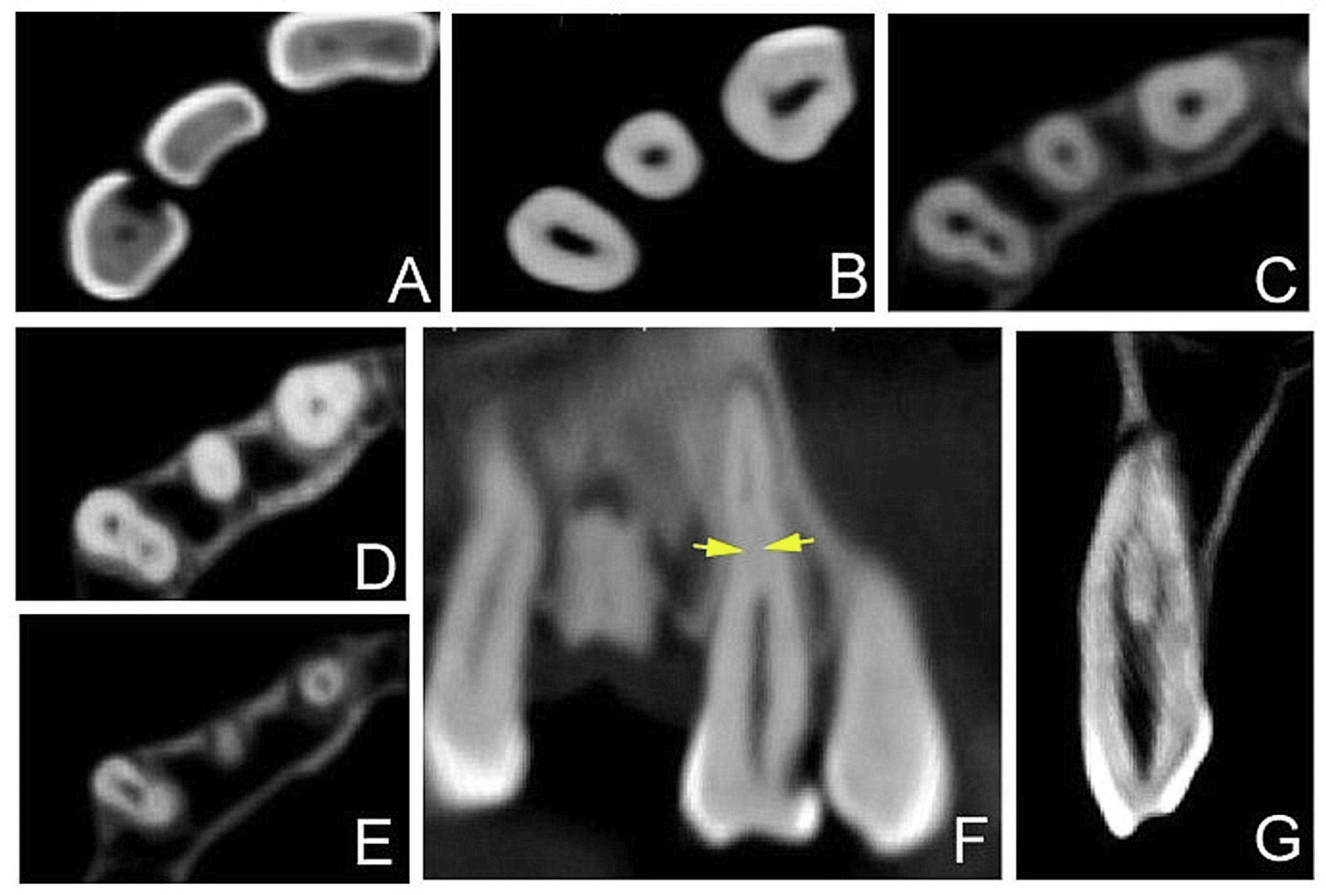
Cone-beam computed tomography images (A-E) Axial view from coronal to apical, with two canals (buccal and lingual appreciable in B and C). (F) Sagittal view (yellow arrow indicating “fast break”. (G) Coronal view with the main canal dividing into two canals and joining apically. Note: The CBCT images were captured from the previously advised full-face CBCT volume, which was used for follow-up of ameloblastoma treatment. Therefore, the image resolution was less than optimal. The patient did not consent to undergo an additional limited field-of-view CBCT specifically of the maxillary canine region.

A diagnosis of pulp necrosis and symptomatic apical periodontitis was established. Non-surgical root canal treatment was planned, and informed consent was obtained. The patient had reported for a follow-up on ameloblastoma treatment, along with a previously advised CBCT volume of the full face (FOV 10x14, 90kV, 8mA, 27s, Planmeca Romexis 5.3.5.80, G-XR-136953, Helsinki, Finland). Hence, before the endodontic treatment was started, the CBCT images in different sections helped us correlate the “fast-break” appearance (on IOPAR), confirming the presence of two canals (Figures 2A-2G).

Local anesthesia (Lignox 2% A, Indoco Remedies Ltd, Mumbai) was administered, and the access cavity was prepared under rubber dam isolation (Figure 1B). Initially, a single canal was located in the cervical third (Labially), and on further exploration with a pre-curved #10 k-file (Mani, Tochigi, Japan), a second canal was located palatally. A radiograph with a mesial shift confirmed the initial working length, as estimated with the electronic apex locator (Root ZX; Morita, Tokyo, Japan) (Figure 1C). The cleaning and shaping were performed using Hyflex rotary files (Coltene/Whaledent Inc., Cuyahoga Falls, OH, USA), and a total of 15 mL of 1% sodium hypochlorite irrigant (Medlise Chemicals, Kerala, India) was used intermittently, along with a 30 Gauge side vent needle (Neoendo, Orikam Healthcare India (P) Ltd., Gurugram, India). The labial and palatal canals were enlarged apically to sizes of #30/0.04 taper and #25/0.04 taper, respectively. Calcium hydroxide intracanal paste (RC Cal, Prime Dental Products Pvt. Ltd., Mumbai, India) medicament was gently syringed into the canal, and access was temporized with Cavit (Cavit G, 3M ESPE, Germany).

After two weeks, the patient reported being asymptomatic, and the tooth was non-tender on percussion. The intracanal medicament was removed using a #20 H-file (Mani, Tochigi, Japan). To ensure the irrigant passes through the fins between the canals, a 3% sodium hypochlorite irrigant was activated with the Endoactivator (Dentsply Maillefer, Ballaigues, Switzerland) for 20 seconds. A final rinse of 17% ethylenediamine tetra-acetic acid (EDTA, Endo-L, Maarc Dental, Maharashtra, India) for one minute, followed by normal saline (Abaris Healthcare Pvt. Ltd., Mehsana, Gujarat, India) was used before obturation. Canals were dried and, after selecting the respective size master cone, the canals were coated with a zinc-oxide eugenol-based sealer (EndoSeal, Prevest DenPro Ltd., Jammu, India), and obturation was done using the warm lateral compaction technique. The access cavity was restored with composite resin (Tetric N-Ceram, Ivoclar Vivadent, Schaan, Liechtenstein) as a post-endodontic restoration. On the immediate post-obturation radiograph, an accessory canal (loop-type) was identified in the middle third. Such a configuration was suggestive of Vertucci type XVII as proposed by Sert and Bayirli [9,12]. However, in the CBCT view, the accessory canal (or third canal) was not appreciable. Hence, the probability of an accessory canal of the loop type could be considered. The patient was unavailable for follow-up due to medically related problems. However, on telephonic review after three years, the patient reported being asymptomatic.

## Discussion

Root canal variations can significantly impact both endodontic diagnosis and treatment outcomes [13] Consequently, practitioners must be familiar with potential deviations from standard root canal anatomy. While maxillary permanent canines typically have a single root and canal, studies have identified anatomical variations across different populations [1,2,4,6,11].

Locating and managing all canals is vital during endodontic treatment. Clinicians must watch for signs of additional canals. When assessing the pre-operative radiograph, consider a careful radiographic evaluation, such as a “ghost” apex, a fast break canal (sudden narrowing or disappearance of the pulp space), and an eccentric canal tracing [14].

Endodontic examination in a clinical setting, potentially aided by magnification under a dental operating microscope (DOM), can result in the detection or suspected presence of additional canals. Locating additional canals may be aided by taking radiographs of different angles with files in place [15]. However, 2D radiographs (IOAPR) may not always accurately reveal multicanal anatomy due to superimposition or angulation errors. Recent improvements in cone-beam and micro-computed tomography have increased reporting of complex root canal anatomy. Apart from being non-invasive CBCT imaging can provide us with the ability to view the tooth and adjacent structures in three dimensions. In the present case, the CBCT images were analyzed from the previously advised full-face CBCT volume, which was used for follow-up of ameloblastoma treatment. Therefore, the image resolution was less than optimal. The patient did not consent to undergo an additional limited field-of-view CBCT specifically of the maxillary canine region. Newer imaging techniques have revealed that existing systems are insufficient for classifying the complexity of many root canal configurations [3].

It is generally accepted that maxillary canines have a single root and a single canal. Caliskan et al.'s study reported that 4.35% of canals were type 3 configurations [10]. Similarly, in most instances, the mandibular canines possess a single root and a single root canal. However, around 15% of cases have been reported to have two canals, which may contain one or two foramina [12,16]. Longitudinally running fins within the canal walls often lead to a network of communication between canals, resulting in the presence of multiple root canals within a single root [15].

A standardized method for classifying accessory canal morphology was suggested by Ahmed et al. in 2018. This classification (using a coding system), in comparison to Vertucci's classification, provides more detailed information about tooth number, root count, root canal configurations, and accessory canal morphologies [17]. In the present case, the accessory canal (loop type) was incidental and was appreciated in the post-obturation radiograph.

While it is possible for a tooth to have three canals, if they are located close together, it may be challenging to instrument all three during root canal treatment. In some cases, only two canals may be identified however, thorough chemo-mechanical procedures may remove the canal debris and facilitate the obturation (warm compaction technique) of the additional canal. This anatomical variant corresponds to type 17 in Vertucci's classification [18].

## Conclusions

Maxillary canines typically have one root and canal. Anatomical variations are common, but the Vertucci type 3 canal configuration is infrequent. While uncommon, these variations are critical to identify for effective root canal treatment. This case study highlights the successful use of CBCT in diagnosing and managing a maxillary canine with a Vertucci type 3 canal configuration and an accessory canal. To manage root canals effectively, it is crucial to have a deep understanding of the different anatomical variations and their management. The findings also underscore the importance of CBCT as a diagnostic tool in endodontics for navigating complex root anatomy.
